# Low preoperative prealbumin predicts the prevalence of complications following liver transplantation

**DOI:** 10.1186/s12876-021-01818-1

**Published:** 2021-05-22

**Authors:** Yuancheng Li, Xingchao Liu, Yan Jiang, Kun Wan, Wei Liu, Yanjiao Ou, Jie Bai, Yuemei You, Feng Hu, Zeliang Xu, Ping Bie, Chengcheng Zhang, Leida Zhang

**Affiliations:** 1 College of Basic Medical Sciences, Third Military Medical University (Army Medical University), Chongqing, China; 2 Sichuan Academy of Medical Sciences and Sichuan Provincial People’s Hospital, Sichuan, China; 3 Department of Hepatobiliary Surgery, Southwest Hospital, Third Military Medical University (Army Medical University), No. 30, Gaotanyan Road, Chongqing, 400038 China; 4 Department of Medical Imagine, People’s Liberation Army of China 949 Hospital, Xinjiang Military Hospital, Xinjiang, China; 5 Department of Surgery and Anesthesiology, Southwest Hospital, Third Military Medical University (Army Medical University), Chongqing, China; 6 Department of Hepatopancreatobiliary Surgery, The Third Affiliated Hospital, Chongqing Medical University, Chongqing, China

**Keywords:** Liver transplantation, Preoperative prealbumin, Postoperative complications, MELD subgroups

## Abstract

**Background:**

As a nutritional index, preoperative serum prealbumin highly correlates with surgical complications. However, the correlation between preoperative prealbumin and postoperative complications remains unclear in liver transplantation (LT).

**Methods:**

A total of 191 patients who underwent LT between 2015 and 2019 were included in the retrospective analysis. According to a cut-off value calculated from a receiver operating characteristic (ROC) curve, the patients were divided into normal and low preoperative prealbumin groups. Univariable and multivariable logistic regression analyses were used to identify independent risk factors for postoperative complications. In addition, patients were divided into subgroups by Model for End-stage Liver Disease (MELD) score, and the association between preoperative prealbumin and postoperative complications was also assessed in each group.

**Results:**

A total of 111 (58.1%) patients were included in the low prealbumin group based on a cut-off value of 120 mg/L. The area under the ROC curve (AUC) was 0.754 (95% confidence interval [CI] 0.678–0.832). Low prealbumin (95% CI 1.51–12.8, *P* = 0.007) was identified as a predictor for postoperative complications based on multivariable regression. In the low and normal prealbumin groups, the prevalence rates of postoperative complications were 27.5% and 8.0% (*P* = 0.003) in the MELD score ≤ 15 subgroup and 53.3% and 20.0% (*P* = 0.197) in the MELD score > 15 subgroup, respectively.

**Conclusions:**

Preoperative prealbumin was associated with postoperative complications in LT, and preoperative nutritional support benefitted postoperative recovery, especially for patients with low MELD scores.

## Introduction

Liver transplantation (LT) is the best method for the radical cure of end-stage decompensated liver disease and malignancy and results in satisfactory replacement of liver function and excellent margins [1–3]. However, postoperative complications remain a concern for short- and long-term survival. Malnutrition significantly increased the risk of postoperative complications [4], while previous research found that preoperative nutritional support reduced complications significantly in abdominal surgery [5]. Patients with end-stage liver diseases frequently suffer from malnutrition with insufficient hepatic synthesis. However, the relationship between nutritional status and post-LT complications has not been thoroughly revealed.

Serum albumin is the most objective and applicable nutritional indicator [6], but it is easily affected by exogenous albumin supplements. In contrast, serum prealbumin, another measurable nutritional indicator, is also able to reflect nutritional status objectively. As a precursor for synthesizing albumin, prealbumin is barely influenced by external supplementation [7]. With a shorter half-life than serum albumin, serum prealbumin is a precise marker to evaluate the severity of liver diseases [8]. Numerous studies have incorporated prealbumin into preoperative nutritional assessments and have used it for surgical risk stratification [9, 10] As for hepatopancreatobiliary diseases, preoperative prealbumin plays a crucial role in predicting postoperative complications, such as for patients undergoing pancreaticoduodenectomy or hepatectomy [11–13]. This relationship was also demonstrated in patients undergoing total knee arthroplasty and kidney transplantation [14, 15]. Moreover, preoperative prealbumin combined with disease severity has been reported to yield better predictions in patients with liver cirrhosis [16]. However, the relationship between preoperative serum prealbumin and post-LT complications has not been demonstrated.

The present study aimed to confirmed the effect of prealbumin on the postoperative complications of LT and whether preoperative nutritional support is highly recommended for LT patients.

## Methods

### Patient selection

In this retrospective study, the clinical characteristics and demographic data of patients in a single-center database were reviewed and selected from the records of the Chinese Liver Transplant Registry (CLTR: http://cltr.cotr.cn). This database contains data from 191 patients who underwent LT from January 2015 to March 2019 in Southwest Hospital. Patients were diagnosed with cirrhosis based on unequivocal imaging results or liver biopsy and symptoms such as ascites and esophageal variceal bleeding. Additionally, the MELD score was used to calculate the severity of liver disease. Postoperative histopathology was used to identify malignant tumors. Perioperative data were collected from 1 to 3 days before surgery until hospital discharge. This retrospective study was conducted in accordance with the Declaration of Helsinki, and no organs from executed prisoners were used for LT.

### Parameter selection

All patients’ medical records were reviewed for a series of clinical and pathological characteristics, including age, sex, body mass index (BMI), history of hepatectomy, HBV infection, American Society of Anesthesiologists (ASA) score, smoking and alcohol abuse. Blood laboratory examinations assessing liver function and nutritional status, such as total bilirubin, aspartate aminotransferase (AST), alanine aminotransferase (ALT), the international normalized ratio (INR), albumin and prealbumin, were performed within a week before the operation. The MELD score was calculated as follows: 3.78 × ln (total bilirubin, mg/dl) + 11.2 × ln (INR) + 9.57 × ln (creatinine, mg/dl) + 6.43.

The prealbumin level was based on the last measurement of the plasma concentrations of prealbumin before surgery. The cut-off value of preoperative prealbumin calculated by ROC curve divided patients into subgroups. Prealbumin was mainly synthesized by liver, having a role of transporting retinol and T4 thyroid hormone. As a negative acute-phase protein, serum level of prealbumin could be decreased in acute infections and inflammatory changes.

### Postoperative assessment

Graft performance was evaluated routinely through daily laboratory examinations after transplantation until hospital discharge. Surgical complications were determined based on clinical symptoms and diagnostic examinations. The postoperative complications of all patients were documented in medical records and research software.

The Clavien-Dindo (CD) classification was used to estimate postoperative complications [17]. We defined CD grade II or greater morbidity as a postoperative complication in this study [18, 19]. The present study recorded all postoperative adverse reactions of the patients and finally documented pneumonia, renal failure, infection, biliary leakage, bleeding, pleural effusion, ascites and liver failure as complications.

### Statistical analysis

Categorical and continuous baseline characteristics and perioperative variables are reported as the quantity, percentage, or median and interquartile range (IQR), as appropriate. Moreover, continuous variables were compared between two groups by Student’s t test or Mann–Whitney rank sum U tests, while categorical variables were compared by the chi-square test or Fisher's exact test. Spearman correlation analysis was used to determine the relationship between preoperative serum albumin and prealbumin. After univariable logistic regression, only variables with *P* < 0.1 were submitted to multivariable analysis. A receiver operating characteristic (ROC) curve and the area under the ROC curve (AUC) were used to confirm the predictive ability of preoperative prealbumin for postoperative complications. Similar to previous studies, this research used the maximum sum of sensitivity and specificity as the best cut-off value.

All tests were 2-tailed, with a *P* value < 0.05 indicating significance. All statistical and graphical analyses were performed using SPSS version 25.0 (IBM SPSS Inc., Chicago, IL, USA).

## Results

### Relationships between preoperative prealbumin and patient characteristics

**Fig. 1:**
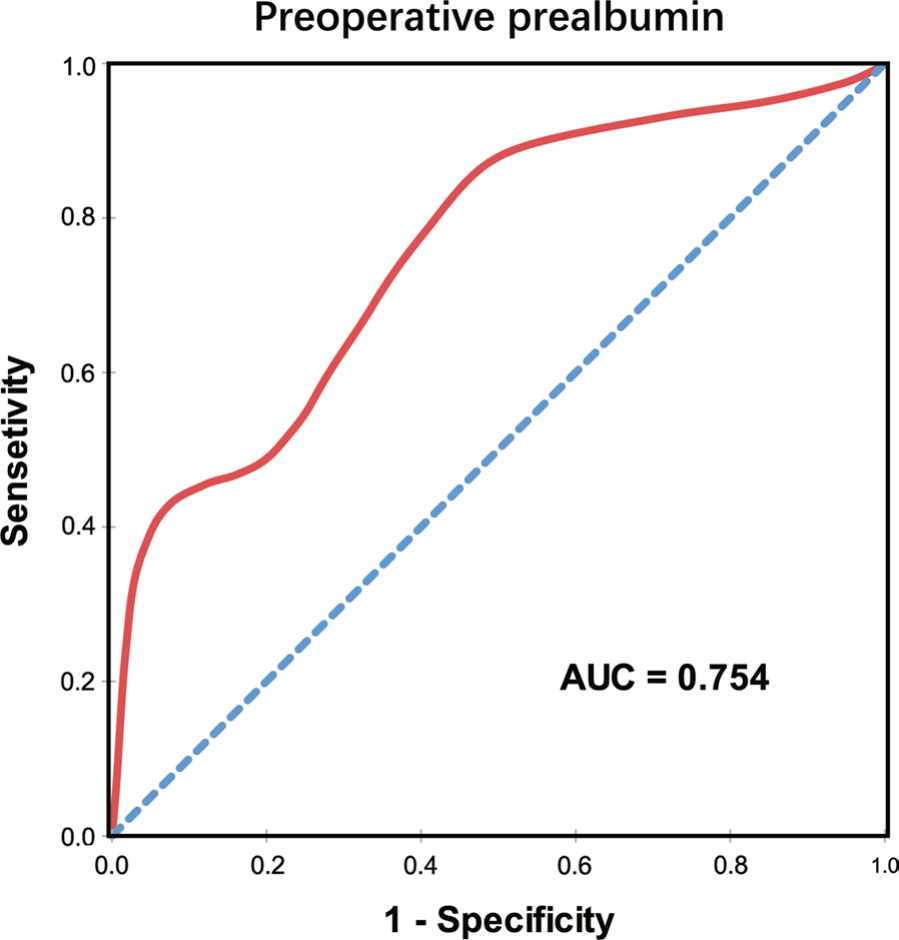
ROC curve for cut-off value of preoperative prealbumin in predicting postoperative complications. The cut-off value of preoperative serum prealbumin to predict postoperative complications was 120 mg/L, according to ROC analysis with AUC of 0.754 (95% CI 0.675–0.832; *P* < 0.001)

A cut-off value of 120 mg/L for preoperative prealbumin calculated by ROC analysis was the optimal criterion, with a sensitivity of 84.7% and a specificity of 54.3%, as shown in Fig. 1. For predicting postoperative complications, the AUC of preoperative prealbumin was 0.754 (95% confidence interval [CI] 0.675–0.832; *P* < 0.001). On the basis of a cut-off value of 120 mg/L, the patients were grouped into low and normal levels of preoperative serum prealbumin. A total of 111 (58.1%) patients with low preoperative prealbumin had a significantly higher rate of postoperative complications than 80 (41.9%) patients with normal preoperative prealbumin within 3 days before surgery.

**Fig. 2:**
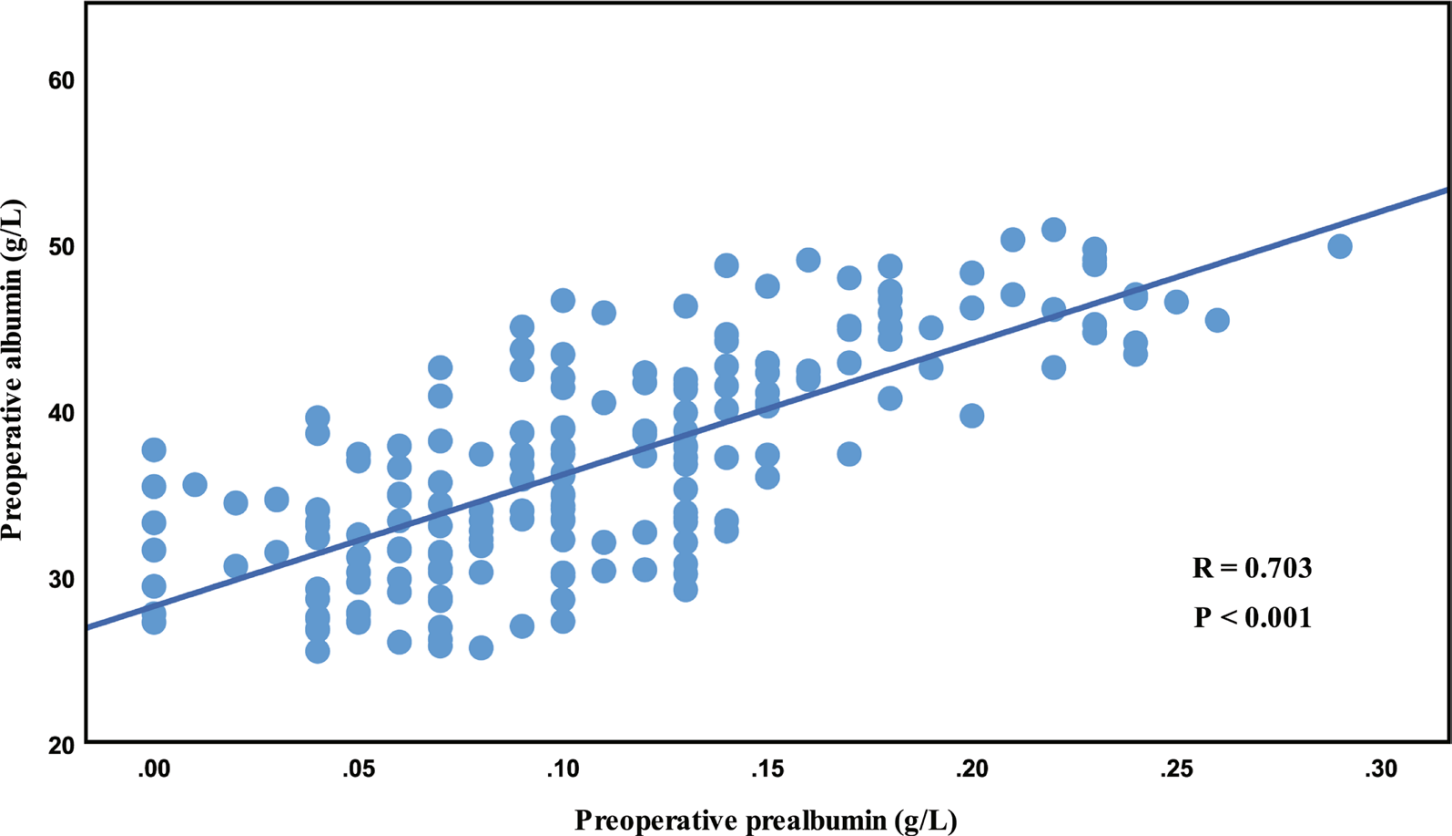
The scatter plot of relationship between preoperative serum albumin and prealbumin

The characteristics of a total of 191 patients are shown in Table 1. Deceased donor LT was performed in all patients, and most of the patients (80.6%) had a history of HBV infection. A total of 161 (84.3%) males and 30 (15.7%) females with a median age of 49 (IQR 42–58) years and a median MELD score of 12.2 (IQR 6.2–20.0) were reviewed. As shown in Fig. 2, Spearman correlation analysis revealed a moderate relationship between preoperative albumin and prealbumin levels (R = 0.703; *P* < 0.001).

**Table 1:** Clinical characteristics of recipients in different prealbumin levels

N (%)	Total (N = 191)	Low prealbumin (N = 111)	Normal prealbumin (N = 80)	*P* value
Age, years^a^	49 (42–58)	49 (42–58)	51 (43–58)	0.557
*Smoking*^*b*^				0.300
Yes	92 (48.2)	57 (51.4)	35 (43.8)	
No	99 (51.8)	54 (48.6)	45 (56.2)	
*Sex*				0.301
Male	161 (84.3)	91 (82.0)	70 (87.5)	
Female	30 (15.7)	20 (18.0)	10 (12.5)	
*Alcohol*				0.323
Yes	60 (31.4)	38 (34.2)	22 (27.5)	
No	131 (68.6)	73 (65.8)	58 (72.5)	
*Hypertension*				0.651
Yes	17 (8.9)	9 (8.1)	8 (10.0)	
No	174 (91.1)	102 (91.9)	72 (90.0)	
*Diabetes*				0.190
Yes	21 (11.0)	15 (13.5)	6 (7.5)	
No	170 (89.0)	96 (86.5)	74 (92.5)	
*Previous hepatectomy*				0.001
Yes	26 (13.6)	7 (6.3)	19 (23.8)	
No	165 (86.4)	104 (93.7)	61 (76.2)	
*HBV infection*				0.016
Yes	154 (80.6)	83 (74.8)	71 (88.8)	
No	37 (19.4)	28 (25.2)	9 (11.2)	
BMI^a^	23.4 (21.0–25.6)	23.4 (21.0–25.9)	22.9 (21.1–25.4)	0.217
MELD score	12.2 (6.2–20.0)	15.2 (5.1–23.6)	11.4 (8.2–13.6)	0.059
Preoperative albumin, g/L	36.3 (31.6–42.1)	33.3 (30.0–37.3)	43.7 (40.0–46.7)	< 0.001
Preoperative prealbumin, g/L	0.10 (0.07–0.14)	0.07 (0.05–0.10)	0.15 (0.13–0.20)	< 0.001
Preoperative ALT, IU/L	43.2 (30.9–72.7)	48.8 (32.2–92.4)	35.6 (27.7–53.0)	0.005
Preoperative AST, IU/L	53.8 (37.8–101.5)	67.6 (47.9–127.7)	37.9 (29.0–46.8)	< 0.001
Preoperative PLT, 10^9/L	72 (47–118)	69 (43–110)	76 (54–134)	0.124
Preoperative TB, umol/L	78.0 (32.9–221.0)	91.3 (33.0–341.0)	68.1 (30.3–102.2)	0.009
Preoperative creatine, umol/L	67.0 (57.0–83.0)	65.2 (53.4–87.3)	71.3 (58.7–82.8)	0.134
Preoperative INR	1.23 (1.11–1.60)	1.33 (1.17–2.16)	1.15 (1.06–1.35)	< 0.001

HBV, hepatitis B virus; BMI, body mass index; MELD, Model for End-stage Liver Disease; ALT, alanine aminotransferase; AST, aspartate transaminase. PLT, platelets; TB, total bilirubin; PT, prothrombin time; INR, international normalized ratio

^a^Values are median (interquartile range)

^b^Values are count (percentage)

### Clinical characteristics

As shown in Table 2, clinical and pathological characteristics were included in the univariable logistic regression analysis, and preoperative MELD score, ASA score, albumin and prealbumin were found to be significantly correlated with the incidence of postoperative complications. After multivariable adjustment, MELD score, preoperative prealbumin and hypertension were shown to be independent predictive factors for postoperative complications. The odds ratios of a MELD score > 15, low levels of preoperative prealbumin and hypertension were 2.86 (95% CI 1.29–6.34), 4.40 (95% CI 1.51–12.80) and 4.09 (95% CI 1.15–14.49), respectively. However, although significant in the univariable analysis, serum albumin did not demonstrate predictive ability in the multivariable analysis.

**Table 2:** Univariate and multivariate analysis for predictors of postoperative complications

Variables	Univariable OR (95% CI)	Univariable *P* value	Multivariable OR (95% CI)	Multivariable *P* value
Age, years	1.00 (0.97–1.03)	0.943		
Male	1.14 (0.49–2.68)	0.764		
BMI	1.06 (0.96–1.16)	0.255		
Smoking	0.62 (0.33–1.18)	0.145		
Alcohol abuse	1.18 (0.60–2.31)	0.638		
Hypertension	2.55 (0.93–7.00)	0.070	4.09 (1.15–14.49)	0.029
Diabetes	0.79 (0.28–2.29)	0.670		
Previous hepatectomy	0.75 (0.29–1.99)	0.568		
HBV infection	0.65 (0.30–1.39)	0.266		
ASA score	4.40 (1.12–17.22)	0.034	1.95 (0.53–7.22)	0.316
Charlson score	1.22 (0.75–2.00)	0.421		
Preoperative MELD > 15	5.47 (2.76–10.81)	< 0.001	2.86 (1.29–6.34)	0.010
Preoperative prealbumin < 120 mg/L	7.38 (3.12–17.49)	< 0.001	4.40 (1.51–12.80)	0.007
Preoperative albumin < 35 g/L	4.21 (2.13–8.31)	< 0.001	1.67 (0.73–3.82)	0.222
Preoperative AST, IU/L	1.00 (1.00–1.00)	0.893		
Preoperative ALT, IU/L	1.00 (1.00–1.00)	0.220		
Preoperative A/G	1.11 (0.61–2.04)	0.735		
Preoperative creatinine, umol/L	1.00 (1.00–1.01)	0.361		
Preoperative PLT, 10^9/L	1.00 (1.00–1.01)	0.905		
Surgical time, hour	1.05 (0.90–1.22)	0.547		

Variables were entered into multivariable logistic-regression analysis, which found significant at *P* < 0.1 in univariable analysis

BMI, body mass index; HBV, hepatitis B virus; ASA, American Society of Anesthesiologists; MELD, Model for End-stage Liver Disease; ALT, alanine aminotransferase; AST, aspartate transaminase; A/G, albumin to globulin ratio; OR, adds ratio; CI, confidence interval

The characteristics of the postoperative complications of the patients are also listed in Table 3, which shows that pneumonia, the number of infections, bleeding and pleural effusion were significantly different between the subgroups. Notably, all the mortality broke out in the low prealbumin group.

**Table 3:** Postoperative complications between low and normal levels of preoperative prealbumin groups during hospitalization

N (%)	Total (N = 191)	Low prealbumin (N = 111)	Normal prealbumin (N = 80)	*P* value
Morbidity	53 (27.7)	46 (41.4)	7 (8.8)	< 0.001
*Infection*				
Pneumonia	42 (22.0)	36 (32.4)	6 (7.5)	< 0.001
Intra-abdominal infection	15 (7.9)	13 (11.7)	2 (2.5)	0.039
*Effusion*				
Pleural effusion	27 (14.1)	22 (19.8)	5 (6.3)	0.008
Ascites	17 (8.9)	13 (11.7)	4 (5.0)	0.177
Intra-abdominal bleeding	8 (4.2)	8 (7.2)	0 (0)	0.022
Biliary leakage	6 (3.1)	5 (4.5)	1 (1.3)	0.394
Renal failure	9 (4.7)	7 (6.3)	2 (2.5)	0.380
Liver failure	3 (1.6)	3 (2.7)	0 (0)	0.266
Mortality	15 (7.9)	15 (13.5)	0 (0)	< 0.001

Clavien-Dindo grade II or greater postoperative complications were defined as morbidity

Values are count (percentage)

### Stratification by MELD score and its correlation with preoperative prealbumin

**Fig. 3:**
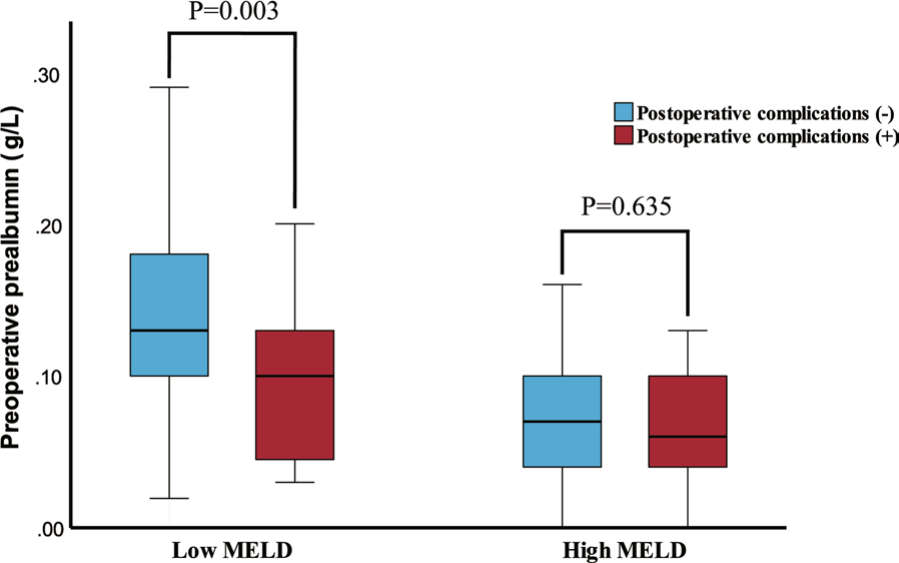
The box plot of distribution of preoperative serum prealbumin with MELD subgroups

To clarify whether preoperative prealbumin was meaningful in patients with different degrees of liver disease severity, patients were divided into low and high MELD subgroups by a cut-off value of 15. A total of 126 (66.0%) patients had a low MELD score, and 65 (34.0%) patients had a high MELD score. According to whether postoperative complications occurred, preoperative prealbumin levels were significantly different between the patients as shown in Fig. 3. However, the distribution of prealbumin among the patients in the high MELD subgroup seemed similar.

Patients in the low MELD subgroup with low preoperative prealbumin, as shown in Table 4, had a higher prevalence of postoperative complications than those with normal preoperative prealbumin (27.5% versus 8.0%, *P* = 0.003). This phenomenon was not apparent in the group of patients with high MELD scores. The rate of postoperative complications among patients with low preoperative prealbumin levels was 53.3%, and although a rate of 20.0% was found among patients with normal preoperative prealbumin levels, the difference was not significant (*P* = 0.197).

**Table 4:** Risk of postoperative complications in different MELD subgroups

MELD ≤ 15	N (%)	Low prealbumin	Normal prealbumin	*P*
	Complications			
	(+)	14 (27.5)	6 (8.0)	0.003
	(−)	37 (72.5)	69 (92.0)	

MELD > 15	N (%)	Low prealbumin	Normal prealbumin	*P*
	Complications			
	(+)	32 (53.3)	1 (20.0)	0.197
	(−)	28 (46.7)	4 (80.0)	

## Discussion

As a widely used indicator, prealbumin can sensitively reflect nutritional status and hepatic synthesis. Once a patient develops an insufficient nutrient reserve or inadequate hepatic synthesis, serum prealbumin decreases obviously. In this state, patients are unable to tolerate surgery and are prone to experience postoperative complications. Therefore, prealbumin might be associated with the risk of postoperative complications. The capacity of preoperative serum prealbumin levels to predict the risk of postoperative complications was demonstrated among patients undergoing LT in this retrospective study. Compared to patients with normal preoperative prealbumin levels, patients with low preoperative prealbumin levels had a higher prevalence of postoperative complications. These results suggested that patients’ nutritional status warrants greater consideration before LT to facilitate an excellent postoperative recovery.

In this study, preoperative serum albumin was not an independent predictor, while preoperative prealbumin showed a strong ability to predict the risk of postoperative complications. Although preoperative albumin is a widely used indicator to evaluate nutritional status and liver function [20, 21], a moderate positive correlation with preoperative prealbumin was also exhibited in scatter plots based on Spearman correlation analysis (R = 0.703, *P* < 0.001). A remarkable finding was that patients frequently received peripheral supplemental infusion of albumin solution due to continuous deterioration of nutritional status and ascites. The level of serum albumin fluctuated with peripheral supplementation, reducing its ability to predict postoperative complications [4]. Although albumin is more widely used in clinical practice, albumin was primarily associated with colloid osmotic pressure and ascites rather than rigorous nutritional support. In addition, a long half-life (17–21 days) led to an inability of serum albumin to respond sensitively to liver damage. In contrast, serum prealbumin was less affected by peripheral supplementation due to its short half-life (2–3 days) and rapid rate of synthesis in response to nutrition supplementation, rendering this parameter a better indicator to reflect the risk of complications [22]. Preoperative prealbumin was shown to be more specific and sensitive in response to nutritional reserve not only in the context of LT but also in hepatectomy, pancreaticoduodenectomy, hemodialysis and kidney transplantation [11, 12, 14].

However, no generally accepted cut-off values are available for serum prealbumin owing to a lack of use and specificity [23]. These results might lead to the inference that the predictive capacity of preoperative prealbumin must be considered separately in different diseases. In this study, the cut-off value of 120 mg/L for preoperative prealbumin was defined by ROC analysis as shown in Fig. 1. The AUC of preoperative prealbumin was 0.754 (95% confidence interval [CI] 0.675–0.832; *P* < 0.001) for calculating the prevalence of postoperative complications. Moreover, low prealbumin was suggested to be an independent predictor for post-LT complications based on multivariable logistic regression analysis (95% CI 1.51–12.8, *P* = 0.007). Therefore, as an indicator of nutritional status and hepatic synthesis, preoperative prealbumin before LT warrants sufficient attention.

Notably, patients with liver diseases frequently suffer from malnutrition because of weakened intestinal peristalsis and anorexia, especially in end-stage liver disease. However, many clinicians prescribe a low-protein diet to prevent hepatic encephalopathy [24]. Acute esophageal variceal bleeding with prolonged fasting is also common in clinical practice. In addition, due to impaired digestive and absorptive abilities, patients are unable to tolerate multiple hospitalizations, examinations and surgical procedures. Malnutrition is closely associated with complications and is considered an independent risk factor for surgical outcomes [25] via numerous mechanisms including impaired fibroblast proliferation and collagen synthesis [26]. In addition, reduced tensile strength and angiogenesis also prolong the inflammatory phase of wound healing. These mechanisms result in delayed healing of the surgical site, intra-abdominal bleeding and a tendency for biliary leakage. Prealbumin (also called transthyretin) has thymus hormone activity and enhances immunity by promoting lymphocyte maturation. In addition to malnutrition, low prealbumin also increases the risks of intra-abdominal infection and pneumonia by impacting lymphocyte function and phagocytic activity [27]. An extremely inadequate nutritional reserve results in a decreased capacity for cell regeneration and protein synthesis. After surgery, patients are prone to developing intra-abdominal bleeding, pleural effusion, bile leakage and infection. Furthermore, the organ burden is increased, resulting in multiple organ dysfunction, such as acute kidney injury and hepatic encephalopathy [28]. Thus, these recipients suffered from high risk of graft dysfunction and even mortality.

The MELD score plays a vital role in calculating the risk of postoperative complications and the hospital length of stay. A number of studies performing subgroup analyses stratified by MELD scores achieved relatively accurate predictions [16, 29]. Extensive literature advocates pre-LT nutritional support for these patients. However, our study suggested that a low prealbumin level was associated with a high risk of complications in the low MELD subgroup. Most patients with higher MELD scores had low prealbumin levels, as shown in Fig. 3. Because of a deteriorated capacity for hepatic protein synthesis, these patients did not have sufficient metabolic activity, thereby decreasing the accuracy of prealbumin for predicting complications. For patients with high MELD scores, malnutrition was only a part of their poor general condition, and transplantation was urgently needed. Nutritional support may not be impactful, and the preoperative delay may not be long enough. In contrast, patients with low MELD scores (< 15) did not need surgery as emergently, and low prealbumin was correlated with postoperative complications (*P* = 0.003). For these patients, nutritional support was vital, and preoperative preparation might be sufficient.

Due to intractable symptoms and multiple organ dysfunction, devoting adequate attention to nutritional assessment and management in patients with high MELD scores is difficult for clinicians. Patients with MELD scores ≥ 15 have been reported to have an overall pretransplant mortality rate of 20 per 100 waitlist-years [3]. Moreover, patients with high MELD scores seem to have impairment of multiple organs, such as the liver, heart and kidneys [30–32]. As a result, numerous factors may increase the prevalence of postoperative complications in these patients. In patients with MELD scores ≥ 20, cardiac insufficiency had a significant influence on the prognosis of transplantation, which was not obvious in patients with MELD scores < 20 [33]. In patients with high MELD scores, other organs may have a greater influence on surgical outcomes, thus masking the role of nutritional status. Therefore, the main objective for patients with high MELD scores is not to improve nutritional status but to undergo transplantation as soon as possible.

However, nutritional supplements cannot be regarded as meaningless. In patients with low MELD scores, pretransplant mortality was lower than 10 per 100 waitlist-years. These patients had an opportunity to receive individualized nutritional management. Previous studies have demonstrated that nutritional support significantly improved serum albumin levels and decreased MELD scores, particularly in Child–Pugh B patients compared to patients with Child–Pugh A cirrhosis [34]. Additionally, with adequate nutritional support, patients’ prealbumin levels reportedly increased by 1–2 mg/dL per day [35]. The cause of low prealbumin levels was not only impaired liver function but also inadequate nutrient intake. Therefore, enteral or parenteral nutrition support was necessary if conditions permitted. Morbidity and the length of hospital stay after surgery were significantly decreased in patients receiving preoperative nutritional support compared to the same parameters in a control group that did not receive nutritional support [5]. Prealbumin was still a prognostic indicator of transplantation. This study showed that patients with normal prealbumin levels were less likely to suffer from complications after surgery, suggesting that patients should be regularly tested for prealbumin while awaiting transplantation. Once patients show low prealbumin levels, especially patients with low MELD scores, which indicate that patients may have more time to wait for a liver, surgeons can initiate measures to improve nutritional status and thus attenuate post-LT complications.

To precisely determine intraoperative risks and postoperative complication rates, preoperative assessment seems especially crucial. In addition to preoperative prealbumin, hypertension was also identified as an independent predictor of morbidity in the present study. Hypertension prior to LT increased the risk of complications, as mentioned in the European Association for the Study of the Liver (EASL) guidelines [36]. Previous studies demonstrated that postoperative complications were related to preoperative clinical markers, including albumin, total bilirubin and ALBI scores [37, 38]. The ALBI score was found to play an important role in evaluating the functional performance of the liver [39, 40]. A poor grade (ALBI grade 3) was significantly associated with postoperative complications and mortality during hospitalization [38]. It is also unclear whether prealbumin could replace albumin and increase the accuracy of these predictors.

Some limitations could not be avoided in our study. First, this was a retrospective study, and excluding all selection biases was difficult. Second, a single center may be subject to institutional problems, which impacts the external effectiveness of research. Most patients had a history of HBV infection. Whether the results of this study can be applied to patients with HCV infection is still questionable. Third, apart from liver cirrhosis, serum prealbumin can be influenced by acute inflammation, hyperthyroidism and nephritic syndrome [7], which affects the accuracy of predictions.

## Conclusion

The present study demonstrated an association between preoperative serum prealbumin and the risk of postoperative complications with a cut-off value calculated from a ROC curve. A more detailed investigation was then conducted among subgroups of patients with different MELD scores. The results suggested that patients with low prealbumin had a higher risk of postoperative complications. Unfortunately, the present study was unable to determine an association between surgical outcomes and dynamic changes in prealbumin. Further studies are needed to determine whether dynamic changes in preoperative prealbumin can serve as an indicator of nutritional support or as a prognostic factor for surgery. In addition, whether prealbumin can replace albumin as a more accurate variable in the context of LT must be confirmed.

## Data Availability

The datasets analyzed during the current study are available from the corresponding author on reasonable request. Since the publication date, all the researchers in hospital or university could send email to corresponding author: zldxngd@163.com. Due to the perioperative data may be updated over time, only three years within the publication date is available.
